# New Appliances and Things Medical

**Published:** 1893-09-30

**Authors:** 


### NEW APPLIANCES AND THINGS MEDICAL.

[All preparations, appliances, novelties, &c., of which a notice is desired, should be sent for the Editor, to care of The Manager, 428, Strand, London, W.C.]

### OLEUSABAN DISINFECTANTS.

We have received from Messrs. Tucker and Co. specimens of their oleusaban eucalyptus disinfectant fluids, powder, and soap. The chief active ingredient of the oleusaban disinfectant is oil of eucalyptus. Thymol and other bodies of that class are also present. It is well known that oil of eucalyptus, which is obtained from various species of eucalyptus, is a powerful antiseptic and disinfectant, being about three times as active as carbolic acid. It is clear, therefore, that as it is very pleasant smelling, it is, if not too dear, certain to be a very popular disinfectant. Messrs. Tucker and Co. are consequently likely to command a large sale, for they have placed before the public so many forms of this disinfectant. Among their list we notice a powder, a fluid, a soap, an ointment, sawdust, spray, dentifrice, tooth powder, bath salts, perfumes, &c. The oleusaban eucalyptus disinfectants are supplied to many public places—as the Glasgow Fever Hospital and Haydcock Union—and they are used by some sanitary authorities and local boards. Messrs. Tucker obtain all the good they can from the eucalyptus species, for they also prepare red gum lozenges, which are excellent as mild astringents for a relaxed throat, and a red gum mixture, useful for diarrhoea.

### WHOLE WHEAT FLOUR WITH HYPOPHOSPHITES.

Messrs. Rawsthorn have forwarded us samples of this flour, which consists of whole meal flour with which hypophosphites of lime, soda, and iron have been incorporated during the process of grinding. It is an ingenious and new idea to mix drugs with our food, but should any one wish to prescribe such a combination he will find it here ready for him.

